# Identifying early permanent teeth caries factors in children using random forest algorithm

**DOI:** 10.3389/fdmed.2024.1359379

**Published:** 2024-04-12

**Authors:** Fatemeh Masaebi, Zahra Ghorbani, Mehdi Azizmohammad Looha, Marzie Deghatipour, Morteza Mohammadzadeh, Mitra Ghazizadeh Ahsaie, Fariba Asadi, Farid Zayeri

**Affiliations:** ^1^ Department of Biostatistics, School of Allied Medical Sciences, Shahid Beheshti University of Medical Sciences, Tehran, Iran; ^2^ Department of Community Oral Health, Dental School, Shahid Beheshti University of Medical Sciences, Tehran, Iran; ^3^ Basic and Molecular Epidemiology of Gastrointestinal Disorders Research Center, Research Institute for Gastroenterology and Liver Diseases, Shahid Beheshti University of Medical Sciences, Tehran, Iran; ^4^ Department of Community Oral Health, Dental School, Zahedan University of Medical Sciences, Zahedan, Iran; ^5^ Department of Biostatistics, School of Public Health, Iran University of Medical Sciences, Tehran, Iran; ^6^ Department of Oral and Maxillofacial Radiology, School of Dentistry, Shahid Beheshti University of Medical Sciences, Tehran, Iran; ^7^ Proteomics Research Center and Department of Biostatistics, School of Allied Medical Sciences, Shahid Beheshti University of Medical Sciences, Tehran, Iran

**Keywords:** dental caries, random forest, permanent teeth, DMFT index, logistic regression

## Abstract

**Introduction:**

Early permanent dental caries can pose a serious threat to oral health in the coming years. This study aimed to investigate the key factors influencing early dental caries in permanent teeth among first-grade Iranian children.

**Methods:**

A cross-sectional study involving 778 randomly selected first-grade children from public schools in Tehran, Iran, was conducted between November 2017 and January 2018. The oral health of the children, evaluated by two trained dentists, was recorded based on the DMFT index. Information on maternal education, gender, dmft index, brushing frequency, dental visits, flossing, and sweet consumption was also collected. The Random Forest method was employed to identify factors associated with early permanent dental caries, and its performance was compared with logistic regression using the Area Under the Curve (AUC) index.

**Results:**

Logistic regression, represented by odds ratios (OR), revealed a significant association between early permanent dental caries and dmft index [OR = 1.13, 95% CI (1.07, 1.20), p-value <0.001], maternal education [OR = 2.04, 95% CI (1.15, 3.62), p-value <0.05], and sweet consumption [OR = 0.59, 95% CI (0.36, 0.98), p-value <0.05]. Random Forest analysis indicated that male gender, higher maternal education, and lower sweet consumption were associated with increased likelihood of being caries-free. Notably, Random Forest demonstrated superior performance (AUC = 0.81) compared to logistic regression (AUC = 0.72).

**Conclusion:**

Early permanent dental caries can be effectively managed by caring primary teeth and reducing consumption of sweets. Maternal education emerged as a pivotal factor in mitigating the risk of early permanent dental caries. Therefore, prioritizing these factors and preventing permanent teeth caries in childhood can be remarkably influential in reducing future caries. The usage of the Random Forest algorithm is highly recommended for identifying relevant risk factors associated with early permanent teeth.

## Introduction

Dental caries represents a significant global oral health concern affecting individuals across diverse age groups. Epidemiological estimates indicate a prevalence of 64.6 million cases in permanent teeth and 62.9 million cases in deciduous teeth worldwide (1). This disease is a multifactorial problem caused by the interaction of specific bacteria in the mouth, a susceptible tooth surface, or a diet high in sugars. Dental caries involves the demineralization and breakdown of the tooth structure, leading to the formation of cavities or holes in the enamel, dentin, and eventually reaching the pulp of the tooth (2). According to the World Health Organization (WHO), dental caries is one of the most prevalent non-communicable diseases among school-aged children, affecting 60%–70% of them in both developing and developed countries (3). The ramifications extend beyond oral health, encompassing pain, infection, impediments to eating and speaking, thereby impacting growth and overall well-being in children. Additionally, untreated caries may disrupt school attendance and performance due to pain and dental appointments.

Dental caries in permanent teeth is commonly evaluated using the DMFT index, which is a valuable tool utilized by dental health professionals to assess oral health status. This index, encompassing decayed, missing, and permanently filled teeth, plays a pivotal role in enhancing children's oral health and alleviating the burden of dental caries in communities (4).

The risk factors associated with dental caries in permanent teeth, as expressed by the DMF index, are multifaceted. Inadequate oral hygiene practices, marked by infrequent brushing and insufficient flossing, contribute to the accumulation of dental plaque and bacteria, fostering tooth decay. High consumption of sugary foods and beverages provides a favorable environment for acid-producing bacteria and leads to an increased risk of dental caries (5). Furthermore, having a higher dmft score in primary teeth can be considered as a risk factor for an increased DMFT score in permanent teeth (6). This may be due to the fact that caries in primary teeth suggests a higher susceptibility to tooth decay, which may persist in permanent teeth. The presence of dental caries in primary teeth can also indicate poor oral hygiene practices, dietary habits, or other factors that may continue to contribute to the development of caries in permanent teeth. Notably, an underexplored risk factor highlighted in the literature is the manifestation of dental caries in permanent dentition during early childhood, specifically at 6–7 years of age. This can be considered as a serious alarm for immediate attention and preventive actions to avoid the development of further caries in the coming years.

In the exploration of factors associated with the DMFT index in children, previous studies have employed diverse statistical methodologies, encompassing traditional univariate approaches and multivariable regression models such as loglinear regression for count responses and logistic regression for binary outcomes when considering the DMFT index. In recent years, advances in statistical methods have led to the utilization of more sophisticated techniques, such as machine learning algorithms, in the field of dental caries research. These methods enable analysts to capture intricate relationships among variables, yielding more precise predictions for diverse health outcomes, including the manifestation of dental caries, and facilitating a more robust identification of associated risk factors. Notably, in the realm of machine learning algorithms, the random forest technique has gained prominence. The random forest approach employs multiple decision trees for prediction, with each tree being trained on a random subset of the dataset. The outcomes from these individual trees are subsequently amalgamated to generate a final prediction. This supervised learning strategy not only enhances predictive accuracy but also provides users with results that are more comprehensible and interpretable (7, 8).

Reviewing the published research in this area demonstrates that there are various studies about the DMFT index and its associated risk factors among school children in different regions of the world. Notably, a study conducted in Korea reported the DMFT index of 2.08 in 12-year-old children. This index was reported as 1.19, 0.70, and 0.70, respectively in the United States, Germany, and Finland. Among 12- and 15-year-old Iranian children, this index was 2.09 and 3.29, respectively (9). In a recent 2021 study involving seventh grade children (age 11–12 years), the researchers found that 48.2% had caries in their permanent teeth, with a mean DMFT of 1.7. This condition was associated with various behavioral and socioeconomic factors, including the screening site, mother's employment, brushing, the status of dental treatment, and visits (10). Furthermore, based on reviewing the statistical methods applied to the published articles, the majority of them have used univariate approaches or simple regression models to discover the relationship between risk factors and dental caries in children. Regarding this, and given the promising attributes of Random Forest, we decided to conduct the present study to assess the performance of random forest for a binary response (DMFT = 0 or DMFT>0) to identify the factors associated with the DMFT index among fist-grade children in Tehran, Iran. Additionally, the accuracy of this advanced method was compared with the binary logistic regression model, which is commonly used in such investigations.

## Methods

### Ethical approval

The present study has been approved by the Ethics Committee of Shahid Beheshti University of Medical Sciences (Ethics code: IR.SBMU.RETECH.REC.1402.136).

### Study design and participants

This cross-sectional study was conducted on 778 first-grade children attending public schools in Pishva and Pakdasht cities, located in the southern regions of Tehran Province, Iran. The study sample was selected using a single-stage cluster sampling technique, wherein first-grade students were randomly chosen from a comprehensive list of both urban and rural students in Pishva and Pakdasht. Subsequently, all first-grade students between the ages of 6 and 7 years were included in the study from November 2017 to January 2018. Inclusion criteria for first-grade children necessitated adherence to specific conditions, including being within the age range of 6 to 7 years, obtaining parental consent, and having no history of congenital or genetic issues. The study consent form provided a detailed explanation of the study's purpose, characteristics, and significance.

### Methods of examination

In the present study, the oral health of children was evaluated by two dentists with specialized training in conducting examinations. To reduce interobserver variability, the dental examiners underwent periodic calibration of their procedures. The examination of the children's teeth was performed using essential tools such as a dental mirror, a round-tip periodontal probe, rubber gloves, cloth or paper hand towels, gauze, and halogen lights. Additionally, standardized WHO forms were employed in collecting information about the children's oral health status and medical records.

### Main outcome and explanatory variables

The primary objective of the study was to assess the occurrence of caries in permanent teeth, with the dependent variable dichotomized as a binary outcome represented by the DMFT index (DMFT = 0 or DMFT>0). In the statistical analysis process, a variety of explanatory variables associated with dental caries were divided into demographic characteristics (including gender and mothers’ educational level; medium (Under 12 years of education) and high (12 or more years of education)) and dental-related information (such as dmft index, having dental visits, frequency of dental flossing and brushing, and sweet consumption frequency).

### Statistical analysis

Frequencies, percentages and box plots were used to summarize and visualize qualitative variables, while means and standard deviations were computed for describing continuous features. The association between the DMFT index and categorical variables was evaluated using the chi-square test, whereas continuous variables were assessed through independent sample *t*-tests.

In order to identify the important variables associated with the DMFT index in first-grade children, the Random Forest method was employed. Based on this statistical approach, the covariate space is recursively partitioned by splitting rules derived from the features. For a given node, the best split is chosen among all possible binary splits obtained from a covariate. Generally, the Random Forest algorithm is one of the most popular approaches, created by drawing bootstrap samples from the original data, so that for each bootstrap dataset, a tree with a selected splitting criterion is constructed. The tree grows up by randomly selecting $q_{0}$ out of q covariates at each node, and when the stopping criterion is reached, the growth ends. Finally, the average results extracted from all trees are used to make an accurate prediction for a new observation.

In this study, the dichotomized DMFT index (consisting of two classes: DMFT>0 or DMFT = 0) was used as the main outcome under study, and the dmft index, gender, tooth brushing, dental flossing, sweet consumption, dental visit, and maternal education level as predictor variables were used to build the Random Forest model.

Variable importance, a key output of the Random Forest method, was assessed through the permutation importance index. In this context, the importance of variables is determined by the permutation importance index. This index determines the importance of variables by assessing the alteration in prediction error during the random permutation process of out-of-bag (OOB) data specific to each variable (7, 8).

The results derived from the Random Forest technique were contrasted with those generated by a dominant parametric methodology, specifically a logistic regression model. It should be noted that the applied Random Forest and logistic regression models were identical in terms of both independent and dependent variables. Furthermore, the strength of the relationship between indicators and the DMFT index was reported using the odds ratio [OR = exp(B)] in the logistic regression model. In assessing predictive efficacy, a comparison of model performance utilizing the Area Under the Curve (AUC) metric was undertaken between the Random Forest and logistic regression models.

The statistical analysis was conducted using R software, version 4.2.2. The Random Forest was constructed based on the default settings, including the number of trees (analyzing 1,000 trees) and the number of variables evaluated to find the best split at each node.

## Results

This investigation encompassed a total of 778 first-grade children, comprising 313 (40.2%) males and 465 (59.8%) females. Within this study, 115 students (14.8%) exhibited at least one decayed permanent tooth (DMFT>0), while 663 subjects (85.2%) remained caries-free (DMFT = 0). Notably, the mean dmft index among children with permanent dental caries was significantly elevated compared to caries-free individuals (7.97 ± 3.44 vs. 6.0 ± 3.88, *p*-value <0.001). Table 1 shows the general characteristics of the participants by DMFT index as the primary outcome. Results indicate significant associations (*p*-value <0.05) between gender, mother's educational level, consumption of sweets, and dental visits, and the dichotomized DMFT index in these children.

**Table 1:** Characteristics of the subjects according to the DMFT index.

Variable	DMF = 0		DMF>0		Total		*p*-value
	*N*	%	*N*	%	*N*	%	
Gender							
Male	276	41.6	37	32.2	313	40.2	0.034
Female	387	58.4	78	67.8	465	59.8	
Dental visit							
No	378	57.0	46	40.0	424	54.5	0.011
Yes	204	30.8	43	37.4	247	31.7	
Brushing							
Less than once a day	234	35.3	37	32.2	271	34.8	0.422
Once or more than once a day	373	56.3	55	47.8	428	55.0	
Flossing							
Less than once a day	552	83.3	79	68.7	631	81.1	0.125
Once or more than once a day	53	8.0	12	10.4	65	8.4	
Consumption of sweets							
Once a day	450	67.9	56	48.7	506	65.0	0.007
More than once a day	158	23.8	36	31.3	194	24.9	
Mother's educational level							
High	533	80.4	69	60.0	602	77.4	0.002
Medium	74	11.2	23	20.0	97	12.5	

In the next step, a multivariable logistic regression model was fitted to investigate the adjusted effect of the covariates on the DMFT index. The results, presented in Table 2, showcase the derived estimates. These estimates illuminate a significant association between mother's educational level, consumption of sweets, and the dmft index with the outcome variable. Specifically, one-unit increase in the dmft index has led to about 13% rise in the odds of dental caries (ie. DMFT>0) in these children (*p* < 0.001). Furthermore, the estimated odds ratio of 0.59 for sweet consumption indicates that children who consume sweets once a day have approximately 41% lower odds of experiencing dental caries compared to their counterparts (*p* < 0.05). Noteworthy is the finding that children with mothers possessing lower levels of education exhibit a 2.04 times higher likelihood of dental caries in permanent teeth compared to those with highly educated mothers (*p* < 0.05).

**Table 2:** Logistic regression analyses of factors associated with the DMFT index.

Variable	Category	B	SE	OR^a^ (95% CI)	*P*-value
dmft		0.126	0.03	1.13 (1.07, 1.20)	<0.001
Gender	Male	−0.275	0.249	0.76 (0.47, 1.24)	0.270
	Female	Reference			
Dental visit	No	−0.296	0.243	0.74 (0.46, 1.19)	0.223
	Yes	Reference			
Brushing	Once or more than once a day	−0.050	0.24	0.95 (0.58, 1.55)	0.842
	Less than once a day	Reference			
Flossing	Once or more than once a day	−0.486	0.363	0.62 (0.30, 1.25)	0.181
	Less than once a day	Reference			
Consumption of sweets	Once a day	−0.525	0.256	0.592 (0.36, 0.98)	0.040
	More than once a day	Reference			
Mother's educational level	Medium	0.712	0.293	2.04 (1.15, 3.62)	0.015
	High	Reference			

^a^ Odds ratio.

**Figure 1:**
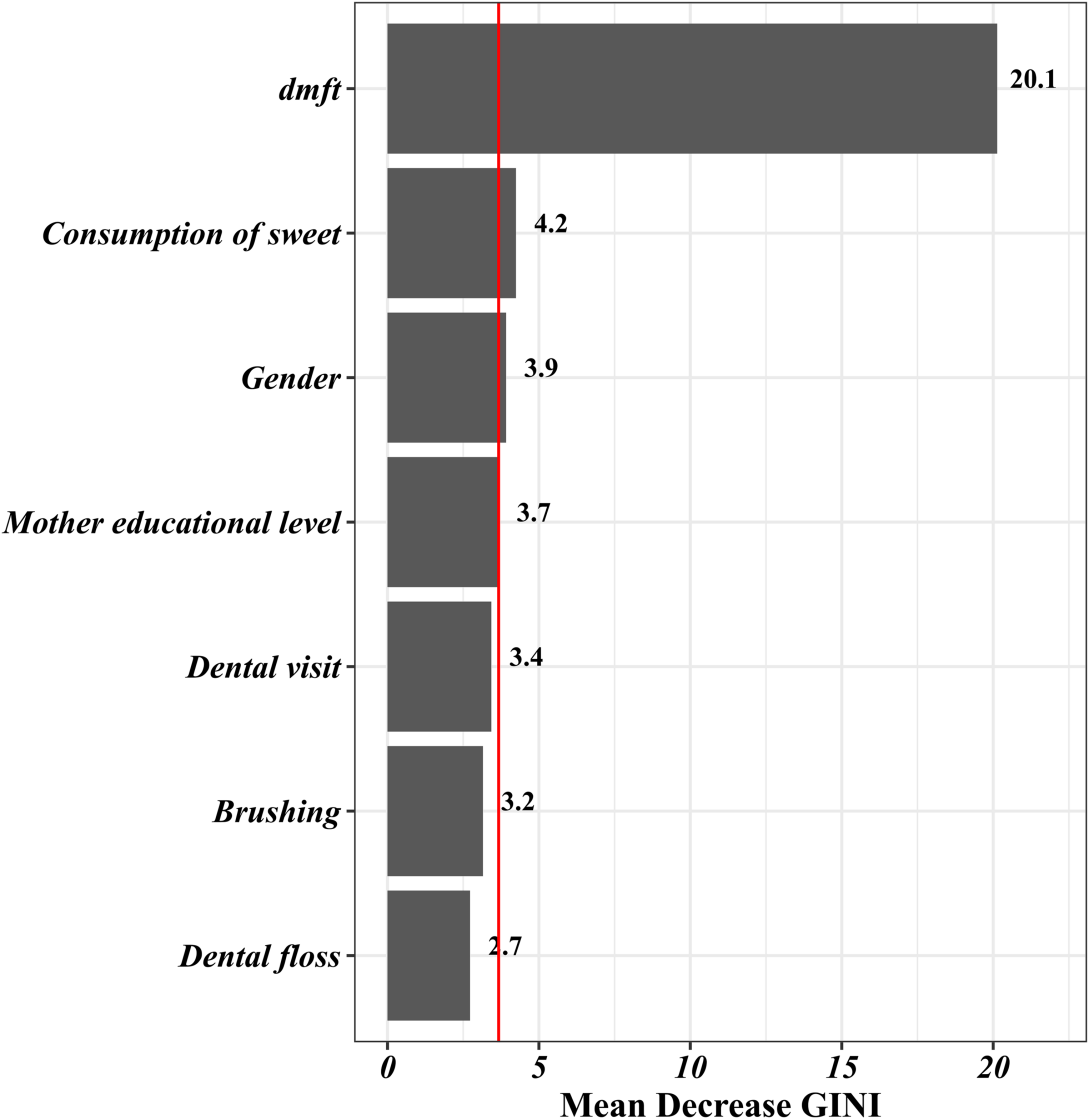
Variable importance of random forest model for predicting the DMFT index in first-grade children.

**Figure 2:**
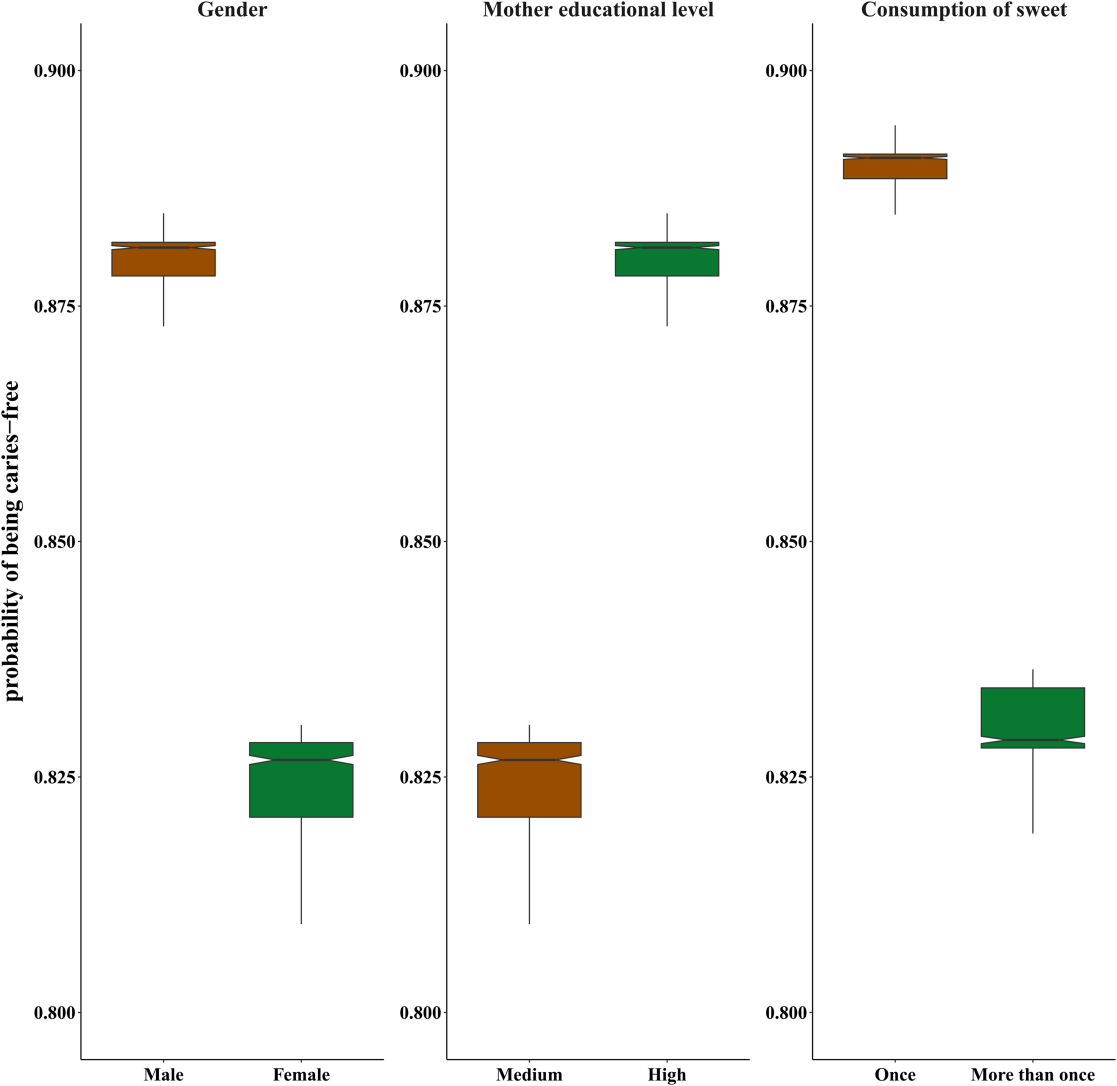
Box plots for probability of being caries-free based on gender, maternal education and sweet consumption.

In the final step, the random forest model was fitted to the data. According to the results of this model, the relative importance of the predictors under study was determined, as illustrated in Figure 1. These results highlight that the dmft index and sweet consumption exhibit the most substantial contributions to the prediction of the DMFT index. Subsequently, the importance of gender and maternal educational level are in the next positions. Furthermore, the probability of the zero DMFT value (being caries-free) for the first three qualitative features extracted from the Random Forest is visualized using a box plot in Figure 2. This plot shows male gender, high maternal education, and a lower tendency to consume sweets can increase the probability of being caries-free. Also, the estimated AUC values for the logistic regression and Random Forest models were 0.72 and 0.81, respectively. This shows a better fit of the random forest model in prediction of the dichotomized DMFT outcome compared to the binary logistic regression model.

## Discussion

Dental caries in permanent teeth, assessed by the DMFT index, constitutes a significant oral health concern across all age groups, with particular relevance to school-aged children. Although, dental caries in permanent teeth is less common in first-grade children, identifying risk factors associated with this problem is crucial for preventive measures and early intervention to reduce the likelihood of developing dental caries in permanent teeth. According to the published evidence, dental caries has detrimental impacts on the quality of life, growth, and academic performance of children. Despite our knowledge about the significant role of identifying dental caries in permanent teeth in early stages, limited studies have been conducted to examine the factors affecting dental caries in permanent teeth. Also, the published articles in this field did not give adequate consideration to capturing the intricate relationship between indicators and outcome in their statistical analysis. This study addresses this gap by employing sophisticated statistical methods to discern crucial factors associated with dental caries in permanent teeth, acknowledging the complexity and nonlinearity of the relationship between the outcome variable and potential predictors. The estimated predictive power indices for the applied models indicated that both of them had acceptable accuracy in predicting dental caries. However, the performance of the random forest model was better than the ordinary logistic regression model. According to our findings, the results of the variable importance analysis using the Random Forest model revealed that dental caries in deciduous teeth and sweet consumption were the predictors with highest relative importance among the assessed indicators. Moreover, gender and maternal educational level had a remarkable influence on the prediction of dental caries in permanent teeth (DMFT). Despite some differences in identifying important variables, the obtained results from the random forest analysis were generally aligned with those found from the logistic regression model.

The obtained results from the present study showed that more than 85% of the first-grade children were caries-free. Consistent with our findings, a study by Masood et al. in 2004 on Malaysian primary school children (aged 6 years) demonstrated a congruent pattern, where the mean (SD) DMFT index was 0.06 (0.306), and 95.4% of children were caries-free. This study underscored a robust association between early caries in permanent teeth at the age of six and the predisposition to future caries (11). In another study by Kamiab et al. in Iran, the researchers stated that 364 (17.9%) of similarly aged children had decayed permanent molars (12). In addition, our findings are similar to the study conducted by Movahed et al. in Iran. They reported a mean of 0.2 for DMFT index in 6-year-old Iranian children. However, our findings are slightly lower than the prevalence of dental caries in permanent teeth reported by two other studies conducted by Kazeminia et al. and Heinrich-Weltzien et al. In a systematic review carried out by Kazeminia et al., the global prevalence of dental decay in permanent teeth was reported to be 53.8% in children. This difference in prevalence of dental decay may be attributed to the diversity in age groups under study in the above-mentioned researches (13). Furthermore, in a study performed by Heinrich-Weltzien et al. on 1,962 Filipino children aged 6–7 year, the prevalence of caries in permanent teeth was reported as 39.7% (14). In another study conducted by Bakhshi et al. in Iran, their findings showed a mean (SD) DMFT of 2.76 (2.67) among 12–15-year-old adolescents (9). This higher DMFT index may be caused by untreated dental caries in permanent teeth in the early stages of childhood. In summary, these findings indicate a markedly higher DMFT index among teenage students compared to younger age groups in Iran, emphasizing the imperative goals outlined by the World Health Organization to reduce the DMFT index among 12- to 15-year old children (15).

Our investigation into demographic factors reveals a noteworthy association between maternal education levels and the prevalence of dental caries in permanent teeth among children. Specifically, our findings indicate that children with mothers possessing lower educational attainment exhibit a substantially higher prevalence of dental caries, with odds more than 2 times greater compared to their counterparts with literate mothers. This finding is supported by the results of various studies across all age groups and different types of teeth. For example, the obtained results from a study, carried out by Nembhwan et al. on 120 mother–child pairs (under 12 years old), revealed that children who had mothers with higher levels of education and occupation had experienced lower scores of DMFT (16). In another study conducted by Borges et al. on 10–17 years old Brazilian students of both genders, a significant correlation between maternal education and dental caries was observed, so that students with mothers who had lower levels of education had a higher prevalence of caries in permanent teeth (17). In a study by Teresa et al. on 7–12 years old students, the results demonstrated that children whose mothers had lower levels of education were more likely to have dental caries (OR = 2.27) (18). The significant association between maternal education and dental caries has also been confirmed by a longitudinal study in Australia. This study, conducted on indigenous children, showed that those with low-educated mothers (less than 10 years of education) tended to have more dental caries compared to those with highly-educated mothers (19). However, in a study by Wu in China, interestingly, multivariable logistic regression analysis showed no relationship between maternal educational level and risk of caries in 12-year-old Chinese students (20). This discrepancy may be attributed to multicollinearity among variables in the employed multivariable model.

The positive impact of maternal education, particularly in oral health education, on reducing dental caries can be effectively managed and controlled. Through targeted oral health educational interventions, there is a proactive opportunity to prevent dental caries. Empowering mothers with essential knowledge and resources enables them to make informed decisions about oral health practices, ultimately leading to a notable decrease in the prevalence and severity of dental caries in both mothers and their children. This assertion is supported by various studies, such as a trial conducted by Deghatipour et al. in 2021 involving 439 mothers observed from pregnancy to 24 months post-delivery to assess interventions aimed at improving children's oral health. Participants were divided into intervention (*n* = 239) and control (*n* = 200) groups. In the intervention group, mothers received heightened awareness through oral health messages delivered via discussions with dentists or face-to-face education by primary healthcare providers. The study concluded that children in the intervention group were more likely to be free from caries by the end of the follow-up period compared to those in the control group (21). Furthermore, in another trial study by Mohebbi et al., Iranian mothers with 1- to 2-year-old children received education on sugar consumption, nighttime feeding practices, and oral hygiene. Results indicated that this intervention significantly improved the oral health of mother-child pairs (22). Therefore, enhancing mothers’ knowledge in oral health can be achieved through various strategies, such as conducting oral health workshops, distributing printed materials, sharing educational videos on social media, integrating oral health education into parenting classes, and providing ongoing support and follow-up sessions. These initiatives play a crucial role in promoting improved oral health outcomes for both mothers and their children by empowering them with essential information and skills to prioritize oral health and implement effective oral hygiene practices at home.

Concerning oral behavior factors, our statistical analysis unveiled two primary findings aligned with existing literature. Firstly, the dmft index (caries index of primary teeth) exhibited a significant impact on the occurrence of caries in permanent teeth among first-grade school children. Specifically, our results indicate that a one-unit increase in the dmft index corresponds to an approximately 13 percent elevation in the odds of developing caries in permanent teeth. In a study conducted by Motohashi et al. on Japanese primary school students, the researchers obtained a significant relationship between the dmft score and caries in permanent teeth. They showed that the dmft score can be considered as a helpful indicator to predict caries in permanent teeth with an acceptable accuracy (6). In a prospective cohort study, which followed Brazilian children from birth to age 12 years, strong evidence was found between the pattern of caries in primary and permanent dentition (23). Additionally, the findings of an eight-year cohort study on 362 Chinese children (at the baseline, they were 3–5 years old) revealed that caries in the primary teeth can predict caries in the permanent dentition with a high degree of accuracy. Precisely, children with caries in their primary teeth were three times more likely to have caries in their permanent teeth (24). In our study, the estimated odds ratio of caries in permanent teeth based on the dmft score was remarkably lower than the studies on Chinese children. This contradiction may be due to the fact that the first-grade school children with a few caries in their permanent teeth were examined.

Second, we found a significant relationship between the occurrence of dental caries in permanent teeth and sweet consumption, so that the children who consume sweets more frequently were more likely to develop dental caries in their permanent teeth. These findings are supported by other studies in this field. For example, in a study conducted by Feldens with the aim of evaluating the association between sugar consumption patterns and permanent dentition caries in 6-year-old children, it was reported that sugar-related behaviors were significantly associated with permanent caries (25). The findings from another study performed by Bassa et al. on 761 school-aged participants showed that the consumption of sweets leads to more than 4 times increase in the risk of dental caries in permanent teeth (26). Additionally, a study by Hong et al. in the UK showed strong evidence for the significant relationship between sweet consumption and decay in permanent teeth in children (27). Furthermore, our study revealed that gender is one of the important demographic factors that affects dental caries. Specifically, our findings indicated that male children in the first grade are at a lower risk of developing dental caries in permanent teeth than females. Several studies have shown a significant association between gender and caries in permanent teeth, with their results indicating a higher risk of caries in females compared to males. For instance, Mubaraki et al. conducted a cross-sectional study in 2022 to explore the prevalence of permanent dental caries among children in Saudi Arabia. Their findings revealed that the overall prevalence of decayed first permanent teeth was 30.5%, highlighting a significant disparity between genders. Specifically, the prevalence of caries in the first permanent teeth was notably higher in females compared to males (28). Additionally, in a longitudinal study conducted in 2016, researchers examined children from Shanghai, China, at the age of 5 years and followed up with them at the age of 12 years. The study revealed that the prevalence of dental caries was 33.9% in boys and 37.9% in girls with mean DMFT scores in permanent teeth of 0.7 ± 0.0 and 0.8 ± 0.1, respectively (29). Also, in a study conducted by Papadaki in 2021, UK children aged 5, 8, 12, and 15 years were considered. Interestingly, no gender disparities in dental caries were found among 5- and 8-year-old children. However, in adolescents aged 12 and 15 years, females had higher DMFT scores than males (30).

In the present study, the advanced method of Random Forest was used alongside the logistic regression model, as a common method, to identify associated factors with early permanent teeth caries. Some unique features of Random Forest method, such as simplicity, versatility, and robust performance, make it more popular compared to other machine learning and deep learning algorithms. Unlike the majority of machine or deep learning models, which act as a “black box”, Random Forest provides a clear understanding of the importance of features in predicting the outcome of our study. This method allows us to accurately investigate all the complex relationships between the indicators and outcome variable. More importantly, Random Forest can be used as a helpful tool to handle imbalanced data by setting the *sampsize* parameter in building each tree in the R software. In other words, the balanced sample solution aims to induce the algorithm to build trees based on a balanced bootstrap sample. This feature was constructive in our study as the utilized data was imbalanced and included a considerable number of zero values. At the same time, there are several potential predictors associated with dental caries, including maternal oral health, socioeconomic backgrounds, and fluoride exposure. Neglecting to account for these factors represents a limitation in our study. Addressing these covariates in our models could enhance the accuracy of models to predict DMFT. For instance, a recent study by Sadegh Zadeh et al. in 2022 applied various machine learning models based on fluoride exposure and other oral factors to predict dental caries in Iranian children. By incorporating these variables, their findings indicated that random forest yielded the highest predictive performance, with an accuracy of 97.4% (31). Likewise, in a study conducted by Ogwo et al. in 2023 involving young adults, the utilization of fluoride, income status, and other oral behavioral factors as covariates in machine learning models like LASSO regression and generalized boosting machines led to improve the accuracy of models. Their results demonstrated that integrating these covariates could boost the AUC of LASSO regression to as high as 93%. Therefore, while our current study incorporated significant influential factors like dmft, recognizing additional variables could further enhance the model's accuracy (32). Despite the appealing features of the Random Forest model in identifying influential factors, it is still rarely used in the field of caries of permanent teeth. For example, in the cross-sectional study conducted by Park et al., machine learning algorithm and logistic regression model were employed to predict early childhood caries in Korean 1- to 5-year-old Korean children. Their findings showed that two methods, with the range of accuracy between 0.774 and 0.785, had an acceptable performance for predicting and identifying associated factors with dental caries (33, 34).

## Conclusion

In summary, our investigation demonstrated that both the Random Forest and logistic regression models exhibit satisfactory accuracy in predicting dental caries. However, a comparative analysis of predictive power indices revealed that the Random Forest model is the preferred choice for predicting dental caries, as it marginally outperformed logistic regression on the examined dataset. Clinically, we found a strong relationship between the educational level of mothers and the occurrence of dental caries in their children. More importantly, we also found that effectively managing dental caries in primary teeth and reducing sweet consumption can significantly decrease the risk of developing caries in permanent teeth, particularly in first-grade children. Additionally, early permanent teeth caries is an important alarm for predicting future caries incidence in adulthood. In light of these findings, it can be conclusively affirmed that early detection of permanent dental caries, coupled with the implementation of preventative measures, is imperative for maintaining optimal oral health. These insights contribute to the broader understanding of the intricate relationships between predictive models, maternal education, early dental care practices, and the long-term implications for oral health.

## Data Availability

The raw data supporting the conclusions of this article will be made available by the authors, without undue reservation.
